# Experimental Study on Bi-Axial Mechanical Properties of Warp-knitted Meshes with and without Initial Notches

**DOI:** 10.3390/ma11101999

**Published:** 2018-10-16

**Authors:** Huiqi Shao, Jianna Li, Nanliang Chen, Guangwei Shao, Jinhua Jiang, Youhong Yang

**Affiliations:** 1 Engineering Research Center of Technical Textiles, Ministry of Education, Donghua University, Shanghai 201620, China; 1142002@mail.dhu.edu.cn (H.S.); nlch@dhu.edu.cn (N.C.); 2 College of Textiles, Donghua University, Shanghai 201620, China; 18317157323@163.com (J.L.); 1179118@mail.dhu.edu.cn (G.S.); 3 Guangdong Polytechnic, Foshan 528041, China; 18621333070@163.com

**Keywords:** warp-knitted mesh, mechanical properties, bi-axial stresses, isotropy, initial notches

## Abstract

Warp-knitted meshes have been widely used for structural reinforcement of rigid, semi-rigid, and flexible composite materials. In order to meet the performance requirements of different engineering applications, four typical warp-knitted meshes (rectangular, square, circular, and diamond) were designed and developed. The mechanical behaviors of these meshes under mono-axial and multi-axial tensile loads were compared. The influence of the initial notch length and orientation on the mechanical performance was also analyzed. The results showed that the biaxial tensile behavior of warp-knitted meshes tended to be more isotropic. The anisotropy level of the diamond warp-knitted mesh was the lowest (λ = 0.099), while the rectangular one was the highest (λ = 0.502). The notch on a significantly anisotropic mesh was propagated along the direction of larger modulus, while for a not remarkably anisotropic mesh, notch propagation was probably consistent with the initial notch orientation. The breaking strength of warp-knitted meshes with the same initial notch orientation decreased with the increase in notch length in both the wale and course directions. For warp-knitted meshes with the same initial notch length, the breaking strength in the wale direction was kept stable at different notch orientations, while that in the course direction decreased remarkably with notch orientation from 0° to 90°.

## 1. Introduction

Recently, the proportion of industrial textiles in whole textile applications has been rapidly increasing in China, especially warp-knitted textiles, which have been widely used in the geotechnical construction, transportation, aerospace, and health fields. Warp-knitted mesh fabrics are one of the most promising products that can be used as structural materials such as geogrid [1], composite reinforcement [2,3], construction concrete reinforcement [4,5], and biomaterial patches [6,7,8], due to their light weight, high porosity, strength, and easy design. Specific geometric morphologies and mechanical properties of warp-knitted meshes are required for different applications. 

There are various warp-knitted mesh structures with corresponding properties. Different shapes can be realized through the design of knitting patterns during the fabricating process, typically rectangle, square, round, diamond, hexagon, etc. With the increase of applications, some research has been done on the mechanical properties of warp-knitted mesh structures. Mohammad et al. [9] compared the mechanical properties of several different types of warp knitting meshes used for hernia repair. Zhang et al. [10,11] analyzed the knitting parameters, such as drawing off density and warp run-in, on the mechanical properties of the diamond warp-knitted mesh fabric. However, these researches are mainly under uniaxial loads, which sometimes cannot sufficiently describe the performance when simultaneously subjected to multi-axial stresses in real conditions. Kawabata developed a linear algebraic method for the evaluation of the biaxial tensile behavior, which could be suitable for both woven and knitted fabrics [12].

Extension and propagation of cracks and defects are important characteristics in designing textile-based structural materials [13]. A lot of researchers have looked at the effects of various defects on the mechanical behavior of textile materials. Minami et al. [13,14] found that the initial cracks have an obvious influence on the tensile strengths of PVC-coated woven fabrics under mono- and bi-axial tensile loads. Bigaud et al. [15,16,17,18] analyzed the failure mechanisms of notched soft composites reinforced by textile structures. Their studies showed that the failure modes depended on the initial crack length, orientation, and loading ratio in the wale and course directions. Luo et al. [19,20] presented a work on the mechanical properties of PVC-coated bi-axial warp-knitted fabric with initial cracks under multi-axial tensile loads. It was found that the crack propagating direction was irrelevant to the initial cracks’ orientations. Triki et al. [21,22] performed tensile central crack tear tests on plain and twill woven fabrics. The tearing energy was proven to be independent of initial crack configurations. However, most of the above mechanisms of crack propagation are developed to explain the mechanical behavior of woven fabrics, woven composites, and biaxial warp-knitted fabrics, which may not suit warp-knitted meshes. Few investigations have been made on the mechanical performance of mesh materials with cracks under bi-axial tensile loads.

In this paper, four typical warp-knitted meshes (rectangular, square, round, and diamond) were prepared that may have great potential in engineering applications. Their mechanical performance was tested under both mono-axial and bi-axial stresses. A comparison of the mechanical behaviors between different knitting structures, as well as the differences under mono-axial and bi-axial tensile test were presented. The influences of initial crack lengths and orientations on the mechanical performance under biaxial stresses were also investigated.

## 2. Experimental

### 2.1. Materials

Polypropylene monofilament for warp-knitted meshes was provided by the State Key Laboratory for Modification of Chemical Fibers and Polymer Materials (Donghua University, Shanghai, China). Its specifications are shown in Table 1.

### 2.2. Fabrication of Warp-Knitted Meshes

**Figure 1:**
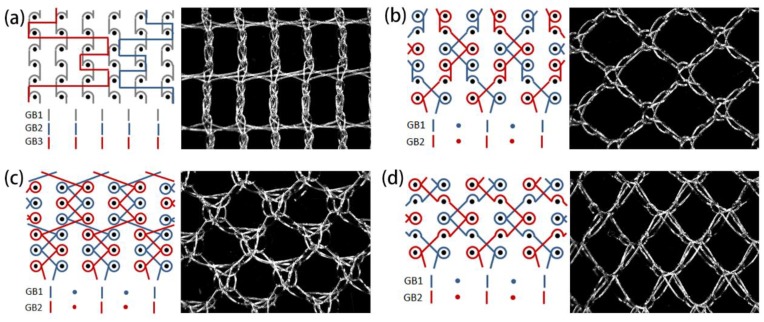
Four typical warp-knitted mesh samples (right side) and their lapping diagrams (left side): (**a**) rectangular, (**b**) square, (**c**) round, and (**d**) diamond-shaped.

Four typical warp-knitted meshes with different mesh shapes were designed and prepared, which were rectangular, square, circular, and diamond-shaped. The lapping diagrams and the loop pictures of the warp-knitted mesh are shown in Figure 1. The device used for this fabrication was an E12 Raschel knitting machine (Changzhou Runyuan Warp Knitting Machinery Co., Ltd., Changzhou, China). The knitting parameters of the warp-knitted meshes are shown in Table 2. The information of fabricated meshes is shown in Table 3, where the density was weighted by a WT2102 electron balance (Changzhou Yi Textile Instrument Co., Ltd., Changzhou, China) with a sample size of 100 mm × 100 mm, the thickness was measured by a YG141N digital fabric thickness gauge (Nantong Hongda Experimental Instrument Co., Nantong, China), and the porosity was calculated with the software Image J (National Institutes of Health, Bethesda, MD, USA).

### 2.3. Testing Conditions

**Figure 2:**
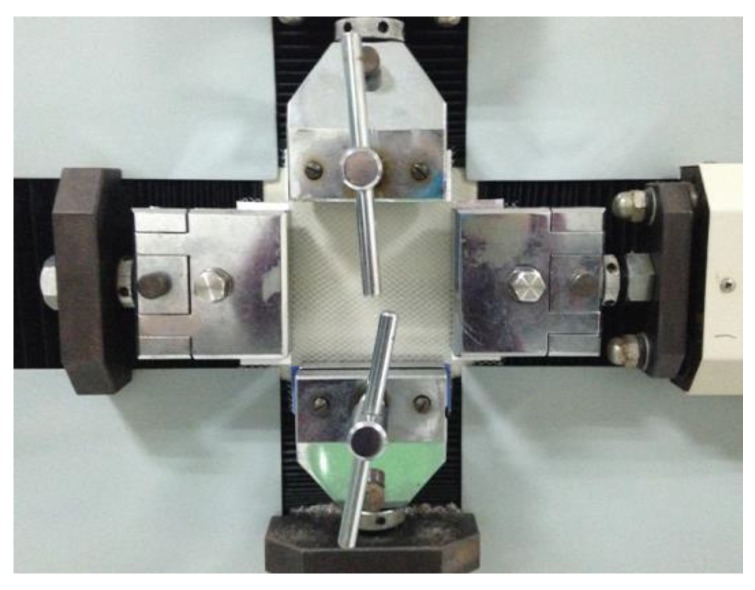
Bi-axial tensile testing device.

The mono-axial tensile test was performed on a WDW-20 universal testing machine (Hualong Testing Instrument Co., Shanghai, China) with a tensile speed of 50 mm/min and a specimen size of 100 mm × 100 mm. Five valid experimental data points each were obtained in the wale (*x*) direction and course (*y*) direction, and the mean value was taken as the experimental result. As shown in Figure 2, the bi-axial tensile testing was carried out on an X‒Y multi-axial electronic strength tester (Darong Textile Machinery Co., Wenzhou, China). The tensile speed in both the *x* and *y* direction was 50 mm/min. Five valid data points were obtained for both the wale (*x*) direction and course (*y*) direction, and the mean value was taken as the experimental result.

**Figure 3:**
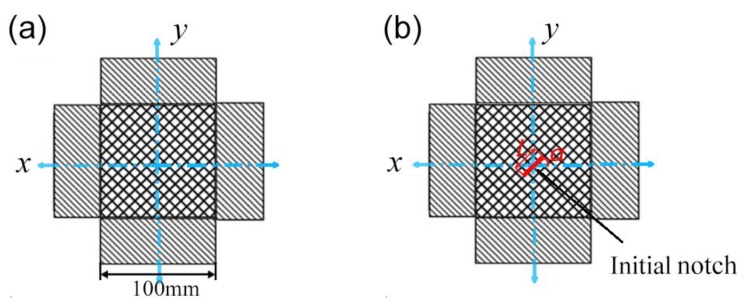
Test samples without initial cracks (**a**) and with initial notch (**b**).

The shape and size of the bi-axial tensile specimen are shown in Figure 3, in which Figure 3a is a non-destructive warp-knitted mesh and Figure 3b is a notched warp-knitted mesh. The load directions were along the wale direction (*x*) and course direction (*y*). The initial notch with length *L* and orientation *α* relating to the *x*-axis was located at the center of the specimen. In order to study the effect of initial notch length on the mechanical properties of warp-knitted meshes, three kinds of notched specimens with different notch lengths (*L* = 1, 3, 5 mesh) and a notch orientation of 0° were prepared. For the influence of crack orientation, three specimens with the same crack length (*L* = 3 mesh) but different crack orientations (*a* = 0°, 45°, 90°) were selected.

## 3. Results and Discussion

### 3.1. Mechanical Behaviors under Mono-Axial and Multi-Axial Tensile Tests

#### 3.1.1. Mono-Axial Tensile Properties

**Figure 4:**
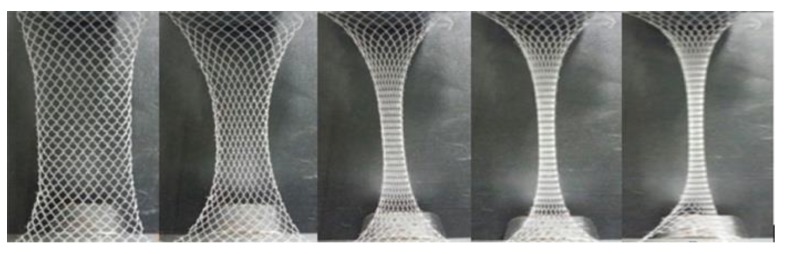
Deformation process of warp-knitted mesh fabric during mono-axial stretching.

Similarities were observed in the deformation process of warp-knitted meshes during a mono-axial tensile test. At the beginning of stretching, the yarns slipped at the crossing points of loop units overcoming small frictions and contact forces [23]. Then the mesh extended along the direction of force, and a large deformation occurred, mainly with the “necking” phenomenon [24]. Finally, the yarn in the mesh unit was straightened, stretched, and slipped until it broke. An example of mono-axial tensile stretching process of diamond warp-knitted mesh is shown in Figure 4.

**Figure 5:**
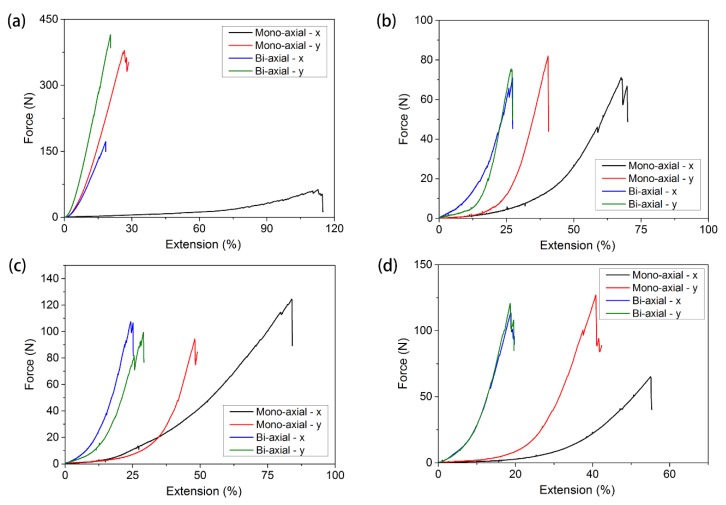
Mono-axial and multi-axial tensile curves of (**a**) rectangular, (**b**) square, (**c**) round, and (**d**) diamond-shaped warp-knitted meshes.

Figure 5 shows the force‒extension curves of four different warp-knitted meshes under mono-axial stress. It can be seen from the diagram that the mechanical properties of warp-knitted meshes in the wale and course directions under mono-axial tension were obviously different. Generally, in a warp knitting structure, the number of yarns in the course direction is less than in the wale direction, so the warp breaking strength and modulus should be larger than the course direction. However, in the circular warp-knitted mesh, the weft breaking strength was greater than that of the warp-knitted mesh, which may be due to the fact that its repeated structure unit was composed of tricot stitch and cord stitch. The loops in the tricot stitch and cord stitch were tightly bound to the underlap. The friction of the underlaps was larger than the wale direction when there was stress in the course direction. The warp strength of the rectangular warp-knitted mesh was about three times that of other structures. Because its structure was three-bar knitting, the plaiting chain and stuffer weft yarn increased the warp binding force. The warp breaking elongation was less than in other structures, since there was only stuffer weft yarn in the course direction. The mono-axial tensile properties of square and diamond warp-knitted meshes were similar, but the breaking strength and elongation of diamond meshes were about 50% higher than those of square meshes.

#### 3.1.2. Bi-Axial Tensile Properties

Warp-knitted meshes are mostly subjected to different directional forces. Therefore, it is of practical significance to study the bi-axial mechanical properties. In the case of bi-axial stretching, the warp-knitted mesh will first quickly overcome the friction of the crossing points between the yarns, then the mesh unit is evenly stretched and expanded along both the wale and course directions until the yarn is stretched to breaking point.

From the bi-axial tensile curves of warp-knitted meshes, as shown in Figure 5, it can be seen that the differences in bi-axial tensile breaking strength of the four meshes were remarkable. The wale and course breaking strength of rectangular warp-knitted mesh were the highest, and the course breaking strength was much higher than that under mono-axial stretching. The wale and course breaking strength of the other three meshes were similar, with the diamond warp-knitted mesh a little higher. Similar to those under mono-axial tension, the course breaking strength of circular warp-knitted mesh under bi-axial was slightly larger than the wale direction. The wale and course elongation of the four warp-knitted meshes under bi-axial stretching were much smaller than those in mono-axial tension.

#### 3.1.3. Mechanical Anisotropy Properties

As shown in Figure 5, after a range of non-linear deformation, which may be about 20–60% of the extension to failure, the relationships between force and extension of warp-knitted meshes become linear in both mono-axial and bi-axial tests. This linear deformation is during the process of stretching the yarns, where most of the applications are working in this range of strain. Therefore, when we assume that the thickness of the meshes does not change after stretching, the asymptotic modulus can be obtained according to the slope of the linear section using the ordinary least squares method:

(1) E=Δσ/Δε.

The results of tensile modulus of four different warp-knitted meshes under mono-axial and bi-axial stresses are shown in Table 4. *E_T_* and *E_L_* are the asymptotic modulus in the wale and course direction, respectively. The orthogonal anisotropy degree λ [9,25] of the warp-knitted mesh can be characterized by the ratio of the modulus in the wale and course directions, where λ = 0 means ideal isotropy, while λ = 1 indicates full anisotropy.

(2) $$\lambda =1-\min(E_{T},E_{L})/\max(E_{T},E_{L}).$$

**Figure 6:**
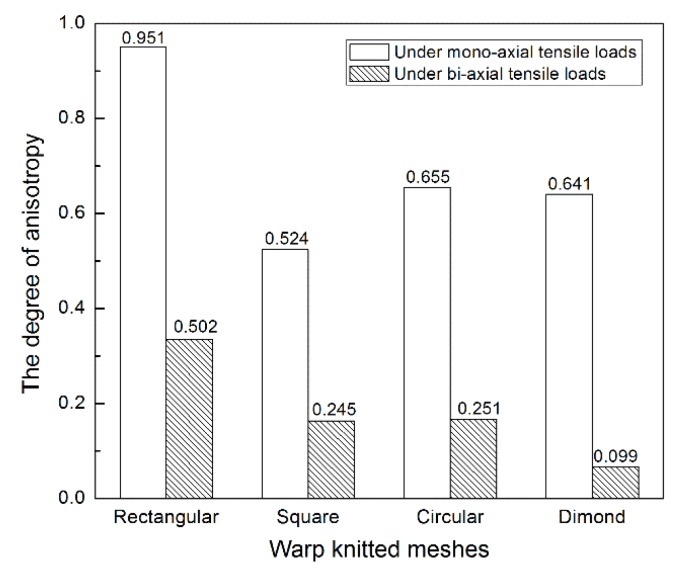
Degree of orthogonal anisotropy for different warp-knitted meshes under mono-axial and bi-axial tensile loads.

Figure 6 shows the anisotropy levels of four warp-knitted meshes under mono-axial and bi-axial stresses. The larger the value of *λ*, the more anisotropic the mesh will be. It is obvious that the level of orthogonal anisotropy under mono-axial stress is much higher than under bi-axial stresses, because the yarn in load-bearing meshes is more well-distributed and uniform when suffering from multi-directional constraints [26]. So the biaxial tensile behavior of warp-knitted meshes tends to be more isotropic. In the case of bi-axial stresses, the least anisotropic mesh was diamond warp-knitted mesh (*λ* = 0.099), which means its mechanical behavior in the wale and course directions tended to be relatively close. The anisotropy levels of square warp-knitted mesh (*λ* = 0.245) and circular warp-knitted mesh (*λ* = 0.251) were similar, slightly higher than that of diamond. The anisotropy level of the rectangular warp-knitted mesh was the highest (*λ* = 0.502), so the mechanical properties would exhibit remarkable anisotropy.

### 3.2. Mechanical Behavior with Initial Cracks

During the knitting process, the phenomena of skipping a stitch, missing a stitch, and the breakage of the knitting yarns when rubbing against metal needles all lead to visible or invisible defects or cracks in the meshes. Furthermore, minor damage may also occur when the meshes are exposed to sharp objects during transportation and utilizations. These kinds of local damage have a significant influence on the performance of composite materials, such as mesh-reinforced concrete [5]. Therefore, research on the influence of cracks on the mechanical properties of warp-knitted mesh is of great significance for various applications.

#### 3.2.1. Notch Propagation Analysis

**Figure 7:**
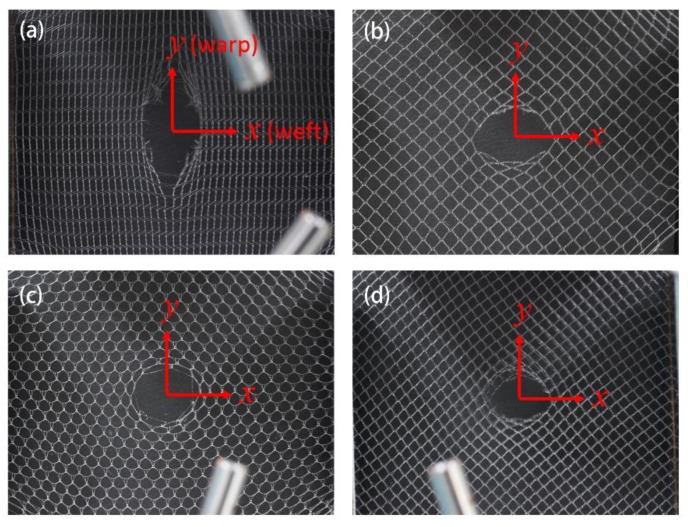
Crack propagation images of (**a**) rectangular, (**b**) square, (**c**) round, and (**d**) diamond-shaped warp-knitted meshes with initial notch of *L* = 3, *α* = 0° under bi-axial tensile loads.

Due to the appearance of notches, the failure mode of warp-knitted mesh under bi-axial stresses seems to be different. During bi-axial stretching of the warp-knitted mesh with initial notch, the stress concentration points were mainly around the notch and near the clamps. The failure process can generally be divided into four stages. Firstly, under the bi-axial loads, the notch in the warp-knitted mesh opened and expanded gradually from the closed state until the edges of the notch were straightened. Then, the deformation was caused by the slippage of the yarns, including the transfer of the top arc to the loop pillar and the extraction of broken yarns from the notch. When the applied loads continued to increase and reached a critical value, the yarns were stretched to breaking point. The notch extended to the next mesh unit. Figure 7 shows the notch propagations of four kinds of warp-knitted meshes with notches (*L* = 3, *α* = 0°) located in the center. By observing the mechanical behavior of different warp-knitted meshes with initial notches, it was found that the direction of notch propagation was related to the anisotropy. For warp-knitted meshes with significant anisotropy, the notches usually propagated along the direction of the larger modulus, such as the rectangular warp-knitted mesh shown in Figure 7a. For a not remarkably anisotropic mesh, notch propagation was probably consistent with the initial notch orientation and the shapes of notch expansion were generally elliptical or circular, as shown in Figure 7b–d.

#### 3.2.2. Effect of Initial Notch Length on Mechanical Properties

**Figure 8:**
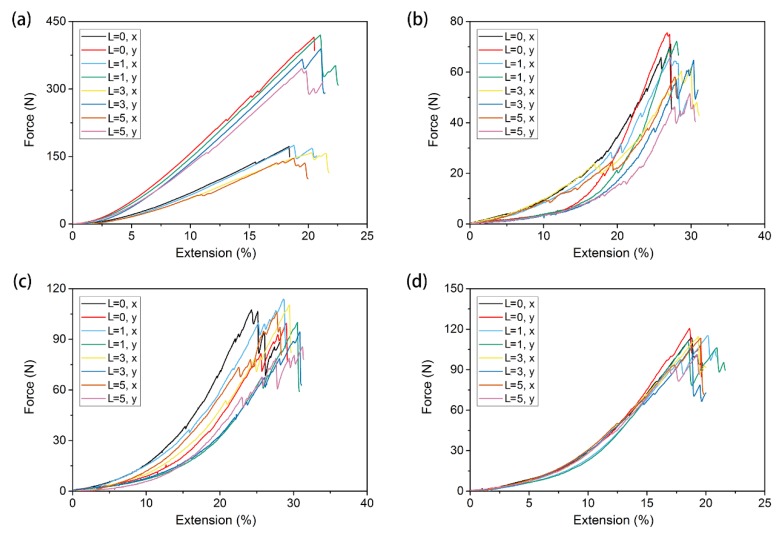
Tensile curves for different initial notch lengths under bi-axial tensile loads: (**a**) rectangular, (**b**) square, (**c**) round, and (**d**) diamond-shaped warp-knitted meshes.

The tensile curves of warp-knitted meshes with the same orientation initial notch (*α* = 0°) and without a notch are shown in Figure 8. In this figure, (a), (b), (c), and (d) correspond to the rectangle, square, circular, and diamond-shaped warp-knitted meshes, respectively. It can be seen from Figure 8 that there are differences in the shape of tensile curves between the specimen with and without notches. The difference is mainly in the tensile modulus and breaking strength, indicating that the existence of an initial notch significantly influenced the mechanical properties of the warp-knitted mesh. The influence was decided by the mesh structures and the notch configurations. The tensile processes of the four warp-knitted meshes were similar. When the yarn broke and the crack expanded, it showed obvious multi-peak characteristics, indicating that other mesh units were not structurally raveled after the breaking notch. This is because the warp-knitted meshes have good resistance to raveling [6]. Comparing the tensile curves of the four warp-knitted meshes, it was found that the influence of initial notches on the tensile modulus of rectangular, square, and circular warp-knitted meshes was more significant than on the diamond mesh.

**Figure 9:**
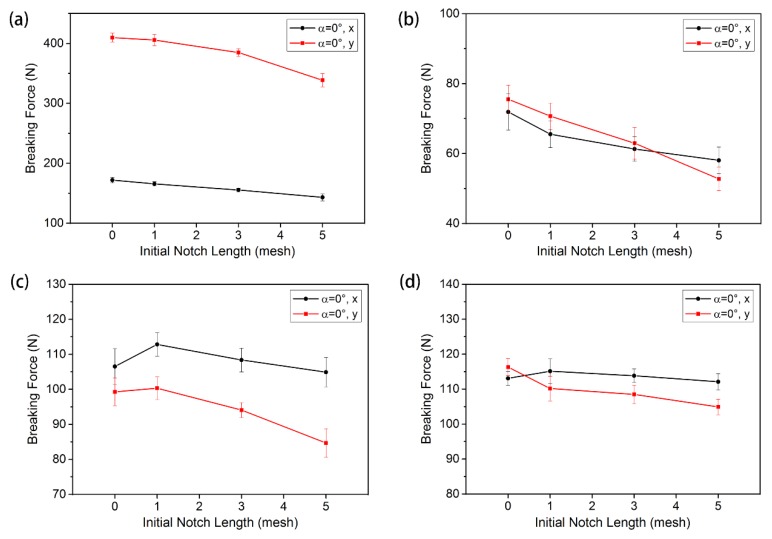
Effect of the initial notch length on the breaking force under bi-axial tensile loads: (**a**) ectangular, (**b**) square, (**c**) round, and (**d**) diamond-shaped warp-knitted meshes.

According to the failure process and tensile curves in Figure 8, when the load reached a critical value, the warp-knitted mesh began to break and the maximum value was called the tensile strength. In this paper, the first load peak was regarded as the breaking strength of the warp-knitted mesh in this direction. Figure 9 shows the bi-axial breaking force of four warp-knitted meshes with different crack lengths. The same trend of the breaking strength and initial notch length was observed in all four warp-knitted meshes, when the notch orientation kept the same (α = 0°). With the increase in notch length, the breaking strength of the warp-knitted mesh decreased in both the wale and course directions. The reason may be that the initial notches were caused by cutting the yarn, which results in a reduction of effective yarn amount within the clamping distance. However, the sensitivity of breaking strength to the notch length was different for knitted structures. Among the four mesh structures, the variation in breaking strength of the diamond warp-knitted mesh with the change of notch length was minimal, as shown in Figure 9d. In the same warp-knitted mesh, the decrease of breaking strength with the notch length in the wale direction was more remarkable than the weft. Because the warp knitting method determines that the overall direction of the yarns is along the wale direction of the fabric [27,28], generally the wale mechanical properties of the warp-knitted fabric are better than the course direction. Therefore, the length of the initial notch had a greater impact on the wale strength. When the notch was very small (*L* = 1), the tensile curves of notched warp-knitted mesh were not significantly different from those without initial notches, as well as the difference of breaking strength. This phenomenon is in accordance with the multi-axial warp-knitted fabrics [20] and woven fabrics [13]. However, with the increase in notch size, the influence on the mechanical properties of the warp-knitted mesh becomes more significant.

#### 3.2.3. Effect of Crack Angle on Mechanical Properties

**Figure 10:**
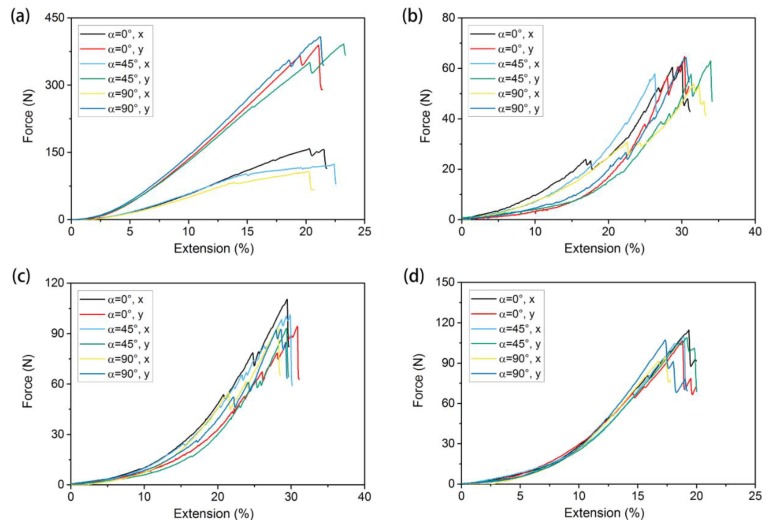
Tensile curves for different initial notch orientations under bi-axial tensile loads: (**a**) rectangular, (**b**) square, (**c**) round, and (**d**) diamond-shaped warp-knitted meshes.

The tensile curves of the warp-knitted meshes with different notch orientations (α = 0°, 45°, 90°) and same notch length (*L* = 3) are shown in Figure 10; (a), (b), (c), and (d) correspond to the rectangle, square, circular, and diamond-shaped meshes, respectively. The tensile curves vary at different orientations from the figure. Therefore, the mechanical performance of a notched warp-knitted mesh is not only affected by the notch length, but also by the notch orientation. The influence also closely depends on the knitting structure. Similar to the phenomenon of notch length, the diamond warp-knitted mesh had the smallest variation with the change in notch orientation, indicating that the performance of a diamond warp-knitted mesh was more stable at different notch orientations.

**Figure 11:**
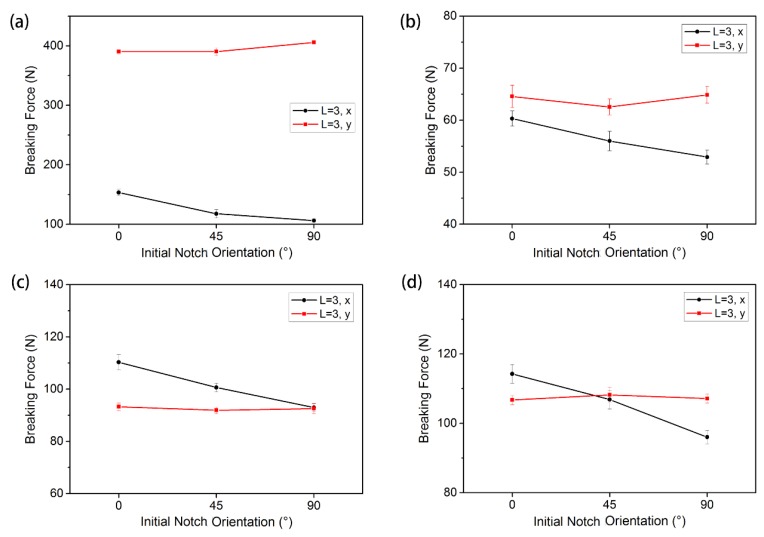
Effect of the initial notch orientations on the breaking force under bi-axial tensile loads: (**a**) rectangular, (**b**) square, (**c**) round, and (**d**) diamond-shaped warp-knitted meshes.

The breaking strengths of warp-knitted meshes with different notch orientation are shown in Figure 11. It can be observed that the mechanical performance trends of the four warp-knitted meshes with the variation of the notch orientation were different between the wale direction (*y* direction) and the course direction (*x* direction). In the wale direction, the change of the breaking strength of the warp-knitted mesh was relatively small, indicating that the effect of notch orientation on the breaking strength of the wale direction was insignificant. When the initial notch length was the same, due to the warp-knitted structure, the number of broken yarns in the wale direction with a notch orientation of 0° and 45° was almost the same. At a notch orientation of 90°, the number of warp broken yarns decreased, but the breaking strength of wale direction will also decrease because of the increase in warp yarn breaking length when under bi-axial stresses. So the breaking strength of the wale direction at the notch orientation of 90° was only slightly larger than that at 0° and 45°. In the course direction, the breaking strength of the warp-knitted mesh decreased linearly with the increase of the notch angle, indicating that the weft breaking strength was more sensitive to notch orientation. Under mono-axial stresses, it was found that the breaking strength of warp-knitted mesh was only sensitive to transverse notches, not to oblique and longitudinal notches [11]. This is completely different from the results under bi-axial stresses. Therefore, the testing methods must be selected appropriately according to the end use conditions.

## 4. Conclusions

In this research, the mechanical properties of four typical warp-knitted mesh fabrics under bi-axial tension were studied, and the effects of different types of prefabricated initial notches on their mechanical performance were analyzed. Based on the results of the experiments and analysis, the following conclusions were obtained:

(1) Warp-knitted meshes presented different mechanical behavior under mono-axial and bi-axial stresses. The meshes under biaxial stresses tended to be more isotropic, and the anisotropy level was decided by the fabric structure. The anisotropy degree of the diamond warp-knitted mesh was the lowest (*λ* = 0.099), while the rectangular one was the most anisotropic (*λ* = 0.502).
(2) The mechanical performance of warp-knitted meshes was affected by notches. The failure mode of the meshes with notches under biaxial stresses was related to the anisotropy level. The notch on a significantly anisotropic warp-knitted mesh (rectangular mesh *λ* = 0.502) usually propagated along the direction of the larger modulus (*E_T_* = 16.845 MPa). For a not remarkably anisotropic mesh (λ = 0.099, 0.245, 0.251), notch propagation was probably consistent with the initial notch orientation.
(3) The breaking strength of warp-knitted meshes with the same initial notch orientation decreased with the increase of notch length in both the wale and course directions. The effect varied according to the fabric structure. The diamond warp-knitted mesh had a minimal effect on the reduction of the mechanical performance. For warp-knitted meshes with the same initial notch length, the breaking force in the wale direction was stable at different notch orientations, while that in the course direction decreased remarkably with notch orientation from 0° to 90°.

## Figures and Tables

**Table 1:** Polypropylene filament specification parameters.

Linear Density dtex	Modulus cN/dtex	Breaking Strength cN/dtex	Breaking Elongation %
169	32.0	5.20	25.4

**Table 2:** Knitting parameters of the warp-knitted meshes.

Mesh	Draw off Density (Loops/cm)	Warp Run-in (mm/480 Courses)		Let-off Ratio
		GB1	GB2	
Rectangular	6.8	2620	920/1280	2.32
Square	6.8	2700	2700	3.89
Circular	6.8	2850	2860	4.06
Dimond	6.8	2840	2840	4.22

**Table 3:** Structural parameters of four typical warp-knitted mesh samples.

Structures	Area Density g/m^2^	Thickness mm	Porosity %
Rectangle	32.2	0.693	72.1
Square	20.8	0.621	74.9
Round	27.6	0.528	67.8
Diamond	21.7	0.488	77.9

**Table 4:** Asymptotic modulus of the warp-knitted meshes.

Warp-knitted Mesh	Mono-Axial Stretch		Bi-Axial Stretch	
	*E_T_*/MPa	*E_L_*/MPa	*E_T_*/MPa	*E_L_*/MPa
Rectangular	13.317	0.654	16.845	8.385
Square	3.298	1.570	3.813	2.879
Circular	3.447	1.190	2.894	3.862
Dimond	3.894	1.400	5.344	4.812
